# Comparative effect of dipeptidyl-peptidase 4 inhibitors on laboratory parameters in patients with diabetes mellitus

**DOI:** 10.1186/s40360-020-00407-4

**Published:** 2020-04-21

**Authors:** Yayoi Nishida, Yasuo Takahashi, Kotoe Tezuka, Hayato Akimoto, Tomohiro Nakayama, Satoshi Asai

**Affiliations:** 1 Division of Pharmacology, Department of Biomedical Sciences, Nihon University School of Medicine, 30-1 Oyaguchi-Kamimachi, Itabashi-ku, Tokyo, 173-8610 Japan; 2 Division of Genomic Epidemiology and Clinical Trials, Clinical Trials Research Center, Nihon University School of Medicine, 30-1 Oyaguchi-Kamimachi, Itabashi-ku, Tokyo, 173-8610 Japan; 3 Division of Laboratory Medicine, Department of Pathology and Microbiology, Nihon University School of Medicine, 30-1 Oyaguchi-Kamimachi, Itabashi-ku, Tokyo, 173-8610 Japan

**Keywords:** Diabetes mellitus, Dipeptidyl-peptidase 4 inhibitor, Sitagliptin, Vildagliptin, Teneligliptin, Alogliptin, Linagliptin

## Abstract

**Background:**

The purpose of this study was to evaluate and compare the effects on laboratory parameters among monotherapy with five DPP-4 inhibitors in patients with type 2 diabetes mellitus (DM).

**Methods:**

We identified cohorts of new sitagliptin users (*n* = 879), vildagliptin users (*n* = 253), teneligliptin users (*n* = 260), alogliptin users (*n* = 237), and linagliptin users (*n* = 180) in patients with type 2 DM. We used a multivariate regression model to evaluate and compare the effects of the drugs on laboratory parameters including HbA1c concentration and serum concentrations of creatinine, estimated glomerular filtration rate, high density lipoprotein, total cholesterol, triglyceride, aspartate aminotransferase, and alanine aminotransferase among the five DPP-4 inhibitors up to 12 months.

**Results:**

Our study showed a favorable effect on HbA1c concentration and a slightly unfavorable effect on serum creatinine concentration in users of the five DPP-4 inhibitors, a favorable effect on lipid metabolism in sitagliptin, vildagliptin, and alogliptin users, and a favorable effect on hepatic parameters in sitagliptin, alogliptin, and linagliptin users, in comparison of the baseline and exposure periods. However, there was no significant difference in mean change in the concentration of any laboratory parameter among the five groups of DPP-4 inhibitor users.

**Conclusions:**

In this study, we showed the effect of five DPP-4 inhibitors on glycemic, renal, and lipid metabolism, and hepatic parameters. DPP-4 inhibitors are well-tolerated hypoglycemic drugs.

## Background

Diabetes mellitus (DM) is a major risk factor for chronic kidney disease, cardiovascular disease, and chronic liver disease, including nonalcoholic steatohepatitis (NASH) and nonalcoholic fatty liver disease (NAFLD) [1–3]. In patients with type 2 DM, alteration of the lipid profile is an important factor in cardiovascular disease [4]. Therefore, it is important to understand the etiology of these complications in patients with DM and to control laboratory parameters associated with renal function, lipoprotein metabolism, and liver function.

By blocking the dipeptidyl-peptidase 4 (DPP-4) enzyme, DPP-4 inhibitors increase insulin secretion by prevention of degradation of incretin hormones including glucagon-like peptide-1 (GLP-1) [2]. DPP-4 inhibitors exhibit different characteristics, including the duration of action, absorption, distribution, metabolism, and elimination. Sitagliptin is eliminated via the kidney, and is mainly excreted in urine as unchanged compound. Therefore, sitagliptin is contraindicated in patients with chronic renal failure [5, 6]. Vildagliptin is excreted by the kidney, but is rapidly converted to an inactive metabolite. Therefore, vildagliptin dosage does not have to be modified in patients with mild renal dysfunction [6]. Teneligliptin is mainly metabolized by cytochrome P450 (CYP) 3A4 and flavin monooxygenases, and approximately 34% is excreted in urine as unchanged compound. Teneligliptin is eliminated via dual hepatic and renal routes, and therefore can be used in patients with renal dysfunction without dose adjustment [7]. Alogliptin is mainly excreted in urine as unchanged compound, and 10% of aloglyptin is metabolized by CYP2D6 and CYP3A4 [8]. Dose adjustment of aloglyptin is not recommended in patients with mild renal dysfunction, but is recommended in patients with moderate to severe renal dysfunction [5, 8]. Linagliptin can be safely used in patients with renal impairment, because, differentiated from other DPP-4 inhibitors, linagliptin is primarily excreted unchanged via an entero-hepatic mechanism [6, 8].

Recently, the pleiotropic effects on several DPP-4 inhibitors have been reported. Sitagliptin administration for 12 weeks was effective in lowering blood pressure, triglyceride (TG), total cholesterol (TC), and alkaline phosphatase concentrations in patients with type 2 DM [9]. A meta-analysis of eight Phase III studies of linagliptin showed that therapy with linagliptin significantly lowered the risk of cardiovascular events versus a comparator. On the other hand, vildagliptin was associated with an increase in liver enzymes. Sitagliptin is contraindicated in patients with chronic kidney disease [5, 6].

Considering the association of DM and various diseases, including chronic kidney disease, cardiovascular disease, dyslipidemia, and chronic liver disease, it is important to investigate which DPP-4 inhibitors influence laboratory parameters other than parameters of glucose metabolism. The aim of this study was to evaluate and compare the effects on laboratory parameters, including renal parameters, lipid metabolism parameters, and hepatic parameters, among monotherapy with five DPP-4 inhibitors, sitagliptin, vildagliptin, teneligliptin, alogliptin, and linagliptin, in patients with type 2 DM.

## Methods

### Data source

This study was a retrospective cohort study utilizing data from the Nihon University School of Medicine (NUSM) Clinical Data Warehouse (CDW) between December 1, 2009 and December 31, 2018. NUSM’s CDW centralizes an order entry database and a laboratory results database, from the hospital information systems at three hospitals affiliated with NUSM, and is described elsewhere [10]. In all databases in NUSM’s CDW, patient identifiers are replaced by anonymized identifiers to protect patient privacy. The data in NUSM’s CDW are mutually linked by anonymized identifiers, and the prescription data of over 0.7 million patients are longitudinally linked with patient demographics, diagnosis, and laboratory data. Several epidemiological studies examining the effects of various drugs on laboratory parameters using NUSM’s CDW have been published [11–13]. The experimental protocol was approved by the Ethical Committee of Nihon University School of Medicine, and the study was conducted in compliance with the Ethical Guidelines for Medical and Health Research Involving Human Subjects of the Ministry of Education, Culture, Sports, Science and Technology and the Ministry of Health, Labour and Welfare, Japan [14].

### Study populations

The subjects of this study were Japanese patients with type 2 DM aged over 20 years who had been newly treated with a DPP-4 inhibitor (sitagliptin, vildagliptin, teneligliptin, alogliptin or linagliptin) listed in Table 1, for at least three months. We identified 2753 patients with type 2 DM treated with sitagliptin (50 mg/day), 1442 with vildagliptin (100 mg/day), 1170 with teneligliptin (20 mg/day), 796 with alogliptin (25 mg/day), and 445 with linagliptin (5 mg/day). We excluded patients who met one of the following six criteria; 1. Patients with severe renal failure (estimated glomerular filtration rate, eGFR < 15), 2. Patients with acute renal failure (ICD10 code: N17), 3. Patients with acute hepatic failure (K72), 4. Patients on dialysis, and patients treated with GLP1 during the study period, 5. Patients who had been treated with other antidiabetic agents and/or lipid-lowering drugs during the exposure period, 6. Patients who had not received regular checks of hemoglobin A1c (HbA1c). After exclusion, the study population consisted of 879 for sitagliptin, 253 for vildagliptin, 260 for teneligliptin, 237 for alogliptin, and 180 for linagliptin (Table 1).

**Table 1:** Numbers of cases of monotherapy with DPP-4 inhibitors

Generic name	Trade name	Dose (daily)	Number of cases of monotherapy
Sitagliptin	Jauvia®, Glactive®	50 mg	879
Vildagliptin	Equa®	100 mg	253
Teneligliptin	Tenelia®	20 mg	260
Alogliptin	Nesina®	25 mg	237
Linagliptin	Trasenta®	5 mg	180

*DPP-4* dipeptidyl-peptidase 4

### Data elements

We collected the demographic data of age and sex, medical histories, and medication of each patients from database to use as a covariates for adjustment. Medical history included the following four diagnoses: 1) cerebrovascular disease (ICD-10 code; I60–69), 2) ischemic heart disease (I20-I25), 3) hyperlipidemia (E78.0-E78.5), and 4) hypertension (I10-I15) during the 365 days before the date of first use of each DPP-4 inhibitor. Medications during the 90 days before the first administration of each DPP-4 inhibitor included the following drugs: 1) oral hypoglycemic drugs, 2) lipid-lowering drugs, 3) antihypertensive drugs, 4) non-steroidal anti-inflammatory drugs (NSAIDs), and 5) steroids.

### Outcomes

The concentrations of HbA1c, serum creatinine, high density lipoprotein (HDL), TC, TG, aspartate aminotransferase (AST), and alanine aminotransferase (ALT) were determined by routine laboratory testing at the hospital of the NUSM. eGFR was calculated using the formula for Japanese subjects specified by the Japanese Society of Nephrology (JSN): eGFR [JSN equation for Japanese] (mL/min/1.73 m^2^) = 194*SCr^-1.094^*Age^-0.287^ (*0.739 if female) [15]. We defined the baseline measurement period, the non-exposure period, as within 3 months before the start of administration of each DPP-4 inhibitor. We defined the exposure period, the outcome measurement period, as between 1 and 3 months (1-3 M) and between 3 and 12 months (3-12 M) after the start of administration of each DPP-4 inhibitor. Laboratory test data for outcome including HbA1c, serum creatinine, HDL, TC, TG, AST, and ALT were collected at the nearest date to the start of DPP-4 inhibitor administration in the baseline period, and at the dates nearest 3 months and 12 months after the start of DPP-4 inhibitor administration in the exposure period. The mean number of exposure days in the 1-3 M period was 56.2 ± 0.5 days for sitagliptin, 56.3 ± 1.0 for vildagliptin, 55.8 ± 1.1 for teneligliptin, 59.0 ± 1.1 for alogliptin, and 53.5 ± 1.1 for linagliptin. The mean number of exposure days in the 3-12 M period was 232.6 ± 6.8 days for sitagliptin, 230.0 ± 12.5 for vildagliptin, 221.7 ± 10.3 for teneligliptin, 224.0 ± 12.6 for alogliptin, and 242.4 ± 13.1 for linagliptin.

### Statistics

We applied a general linear model for continuous data (age and baseline values of laboratory parameters) and chi-squared test for categorical data for comparing the differences in baseline characteristics among the five DPP-4 inhibitors, sitagliptin, vildagliptin, teneligliptin, alogliptin, and linagliptin. We applied a mixed linear model, which was adjusted for age and sex, for the assessment of the differences in mean values of laboratory parameters between the baseline and exposure periods. A multiple-comparison test (Dunnett’s post-hoc analysis) was used to analyze the differences in least square means between the baseline and exposure periods. This study was a retrospective observational study with repeated measures data of non-randomized subjects, which had inherent issues of selection bias and confounding factors. Therefore, we used an adjusted mixed linear model to assess the differences in mean changes in values of laboratory parameters among the five DPP-4 inhibitors. To adjust the model for potential confounding factors, we used the following background variables which were unbalanced among the five DPP-4 inhibitors; time, age, sex, medical history in baseline period including ischemic heart disease and hypertension, medication in baseline period including hypoglycemic drugs and lipid-lowering drugs, and baseline concentration of HbA1c. In addition, the baseline concentrations of creatinine, HDL, AST and ALT were included in the covariates in each analysis of creatinine, HDL, AST and ALT, because differences in baseline values might influence these parameters. All reported *p*-values of less than 0.05 were considered to indicate statistical significance. All statistical analyses were performed using SAS software, version 9.4 (SAS Institute Inc., Cary, NC).

## **Results**

Table 2 shows the prevalence of treatment with antidiabetic drugs during the baseline period. The percentage of patients who had not received any therapy with antidiabetic drugs before the initiation of DPP-4 inhibitors was 28.6% for the sitagliptin group, 39.1% for vindagliptin, 55.8% for teneligliptin, 39.2% for alogliptin, and 49.4% for linagliptin.

**Table 2:** Antidiabetic drugs prior to administration of DPP-4 inhibitors

Antidiabetic drugs	Sitagliptin	Vildagliptin	Teneligliptin	Alogliptin	Linagliptin
Insulin	145 (16.5%)	26 (10.3%)	55 (21.2%)	8 (3.4%)	30 (16.7%)
Sulphonylurea	333 (37.9%)	69 (27.3%)	36 (13.9%)	71 (30%)	30 (16.7%)
Biguanide	275 (31.3%)	63 (24.9%)	36 (13.9%)	49 (20.7%)	34 (18.9%)
Alpha-glucosidase inhibitor	189 (21.5%)	43 (17%)	22 (8.5%)	46 (19.4%)	15 (8.3%)
Thiazolidinedione	102 (11.6%)	21 (8.3%)	7 (2.7%)	51 (21.5%)	4 (2.2%)
Glinide	75 (8.5%)	21 (8.3%)	10 (3.9%)	11 (4.6%)	15 (8.3%)
SGLT2 inhibitor	5 (0.6%)	2 (0.8%)	10 (3.9%)	0 (0%)	3 (1.7%)
Nothing	251 (28.6%)	99 (39.1%)	145 (55.8%)	93 (39.2%)	89 (49.4%)

*DPP-4* dipeptidyl-peptidase 4, *SGLT2* sodium glucose co-transporter 2

Table 3 shows the baseline characteristics of each DPP-4 inhibitor group in our study. Mean age was 63.1 ± 0.4 in sitagliptin users, 64.2 ± 0.8 in vildagliptin, 66.0 ± 0.8 in teneligliptin, 63.5 ± 0.8 in alogliptin, and 67.2 ± 0.9 in linagliptin. The percentage of females was 317 (36.1%) in sitagliptin users, 79 (31.7%) in vildagliptin, 87 (33.5%) in teneligliptin, 72 (30.4%) in alogliptin, and 58 (32.2%) in linagliptin. There were significant differences in mean age and the proportions of patients with a medical history of ischemic heart disease and hypertension, and treatment with antidiabetic drugs and lipid-lowering drugs among the five groups of DPP-4 inhibitor users.

**Table 3:** Baseline characteristics of users of DPP-4 inhibitors

Variables	Sitagliptin	Vildagliptin	Teneligliptin	Alogliptin	Linagliptin	*p*-value
	*n* = 879	*n* = 253	*n* = 260	*n* = 237	*n* = 180	
Age (years, mean ± SE)	63.1 ± 0.4	64.2 ± 0.8	66 ± 0.8	63.5 ± 0.8	67.2 ± 0.9	0.0002*
Sex (female)	317 (36.1%)	79 (31.2%)	87 (33.5%)	72 (30.4%)	58 (32.2%)	0.3791
**Medical History**						
Cerebrovascular disease	24 (50%)	7 (14.6%)	7 (14.6%)	6 (12.5%)	4 (8.3%)	0.9963
Ischemic heart disease	74 (8.4%)	29 (11.5%)	14 (5.4%)	29 (12.2%)	20 (11.1%)	0.038*
Dyslipidemia	95 (10.8%)	44 (17.4%)	28 (10.8%)	27 (11.4%)	23 (12.8%)	0.0688
Hypertension	78 (8.9%)	34 (13.4%)	38 (14.6%)	26 (11%)	34 (18.9%)	0.0008*
**Medication**						
Antidiabetic drug	628 (71.4%)	154 (60.9%)	115 (44.2%)	144 (60.8%)	91 (50.6%)	<.0001*
Lipid-lowering drug	377 (42.9%)	115 (45.5%)	101 (38.9%)	127 (53.6%)	76 (42.2%)	0.0138*
Antihypertensive drug	513 (58.4%)	156 (61.7%)	154 (59.2%)	157 (66.2%)	114 (63.3%)	0.2087
NASID	177 (20.1%)	44 (17.4%)	54 (20.8%)	45 (19%)	33 (18.3%)	0.8433
Steroid	67 (7.6%)	21 (8.3%)	33 (12.7%)	22 (9.3%)	22 (12.2%)	0.0695

*DPP-4* dipeptidyl-peptidase 4, *SE* standard error, *NSAID* non-steroidal anti-inflammatory drug

*p < 0.05 (among five DPP-4 inhibitors)

Table 4 shows the unadjusted and adjusted baseline concentrations of laboratory parameters. There were significant differences in the baseline concentrations of HbA1c, serum creatinine, HDL, AST, and ALT and eGFR among the five groups of DPP-4 inhibitor users.

**Table 4:** Baseline values of laboratory parameters

Laboratory parameters	Sitagliptin	Vildagliptin	Teneligliptin	Alogliptin	Linagliptin	*p*-value
	mean (95%CI)	mean (95%CI)	mean (95%CI)	mean (95%CI)	mean (95%CI)	
HbA1c (%)	7.8 (7.7, 7.9)	7.8 (7.6, 7.9)	7.8 (7.7, 8)	7.4 (7.2, 7.6)	7.4 (7.3, 7.6)	<.0001*
Creatinine (mg/dL)	0.8 (0.77, 0.82)	0.89 (0.85, 0.93)	0.94 (0.9, 0.98)	0.82 (0.78, 0.87)	1.04 (0.99, 1.09)	<.0001*
eGFR (mL/min/1.73m^2^)	74 (72.6, 75.5)	70.3 (67.6, 73.1)	66.3 (63.7, 68.9)	72.5 (69.7, 75.3)	61.8 (58.6, 65)	<.0001*
HDL (mg/dL)	51.3 (50.3, 52.3)	47.3 (45.4, 49.1)	50.1 (48.3, 52)	47.5 (45.6, 49.5)	48.9 (46.7, 51.1)	0.0002*
TC (mg/dL)	192.3 (189.6, 194.9)	188.7 (183.7, 193.7)	190.1 (185.2, 194.9)	188 (182.6, 193.4)	189.3 (183.5, 195.1)	0.5242
TG (mg/dL)	145 (139.1, 151)	160.3 (148.9, 171.7)	151.3 (140.5, 162)	155 (143.3, 166.6)	146.2 (133.2, 159.2)	0.1483
AST (U/L)	27.8 (26.5, 29.1)	30.3 (27.9, 32.7)	29.8 (27.5, 32.2)	29.9 (27.4, 32.4)	24.7 (21.9, 27.5)	0.0124*
ALT (U/L)	30.4 (28.7, 32.1)	32.3 (29.1, 35.6)	30.2 (27.1, 33.3)	34.7 (31.4, 38.1)	26.2 (22.4, 29.9)	0.0157*

*p < 0.05 (among five DPP-4 inhibitors)

*HbA1c* hemoglobin A1c, *eGFR* estimated glomerular filtration rate, *HDL* high density lipoprotein, *TC* total cholesterol, *TG* triglyceride, *AST* aspartate aminotransferase, *ALT* alanine aminotransferase, *CI* confidence interval

Table 5 shows the least square mean concentrations of laboratory parameters during the study period. Compared with baseline, HbA1c concentration was significantly decreased in the exposure period in all DPP-4 inhibitor users. Serum creatinine concentration was significantly increased in the exposure period in all DPP-4 inhibitor users. eGFR was significantly decreased in the exposure period in patients with sitagliptin, vildagliptin, teneligliptin, and linagliptin, and significantly decreased during 3 months in patients with alogliptin. Serum HDL concentration was significantly decreased in the exposure period in patients with sitagliptin and vildagliptin. Serum TC concentration was significantly decreased during 3 months in patients with sitagliptin, vildagliptin, and alogliptin. Serum TG concentration was significantly decreased during 3 months in patients with sitagliptin, vildagliptin, and alogliptin. Serum AST concentration was significantly decreased during 3 months in patients with alogliptin. Serum ALT concentration was significantly decreased in the exposure period in patients with sitagliptin, alogliptin, and linagliptin,

**Table 5:** Relationship between treatment duration and laboratory parameters

Laboratory parameters	Drugs	Time point	N	Unadjusted	*p*-value	^a^Adjusted	*p*-value
				LS Mean (95%CI)		LS Mean (95%CI)	
HbA1c (%)	Sitagliptin				<.0001		<.0001
		baseline	879	7.8 (7.7, 7.9)	reference	7.8 (7.8, 7.9)	reference
		0-3 M	835	7.4 (7.3, 7.4)	*	7.4 (7.3, 7.5)	*
		3-12 M	208	7.2 (7, 7.3)	*	7.2 (7.1, 7.3)	*
	Vildagliptin				<.0001		<.0001
		baseline	253	7.8 (7.6, 7.9)	reference	7.7 (7.6, 7.9)	reference
		0-3 M	241	7.2 (7, 7.3)	*	7.1 (7, 7.3)	*
		3-12 M	64	7.1 (6.9, 7.3)	*	7 (6.8, 7.2)	*
	Teneligliptin				<.0001		<.0001
		baseline	260	7.8 (7.7, 8)	reference	7.9 (7.7, 8)	reference
		0-3 M	237	7.2 (7.1, 7.4)	*	7.2 (7.1, 7.4)	*
		3-12 M	95	6.9 (6.7, 7.1)	*	6.9 (6.7, 7.1)	*
	Alogliptin				<.0001		<.0001
		baseline	237	7.4 (7.3, 7.5)	reference	7.4 (7.3, 7.6)	reference
		0-3 M	216	7 (6.8, 7.1)	*	7 (6.9, 7.1)	*
		3-12 M	61	6.8 (6.6, 7)	*	6.8 (6.6, 7)	*
	Linagliptin				<.0001		<.0001
		baseline	180	7.4 (7.3, 7.6)	reference	7.4 (7.3, 7.6)	reference
		0-3 M	176	7 (6.8, 7.1)	*	6.9 (6.8, 7.1)	*
		3-12 M	58	6.8 (6.6, 7)	*	6.8 (6.6, 7)	*
creatinine (mg/dL)	Sitagliptin				<.0001		<.0001
		baseline	831	0.8 (0.78, 0.82)	reference	0.76 (0.75, 0.78)	reference
		0-3 M	795	0.82 (0.8, 0.84)	*	0.79 (0.77, 0.8)	*
		3-12 M	198	0.86 (0.83, 0.88)	*	0.82 (0.8, 0.85)	*
	Vildagliptin				0.035		0.0375
		baseline	233	0.89 (0.84, 0.94)	reference	0.84 (0.79, 0.89)	reference
		0-3 M	222	0.91 (0.86, 0.97)		0.87 (0.81, 0.92)	
		3-12 M	60	0.93 (0.87, 0.99)		0.88 (0.82, 0.94)	
	Teneligliptin				<.0001		<.0001
		baseline	259	0.94 (0.88, 1)	reference	0.88 (0.82, 0.94)	reference
		0-3 M	239	0.98 (0.91, 1.04)	*	0.92 (0.86, 0.98)	*
		3-12 M	98	1.05 (0.98, 1.12)	*	0.99 (0.92, 1.06)	*
	Alogliptin				0.0001		0.0001
		baseline	226	0.82 (0.79, 0.86)	reference	0.77 (0.74, 0.8)	reference
		0-3 M	205	0.85 (0.82, 0.88)	*	0.8 (0.77, 0.83)	*
		3-12 M	62	0.86 (0.82, 0.9)	*	0.81 (0.77, 0.85)	*
	Linagliptin				0.002		0.0021
		baseline	174	1.04 (0.95, 1.12)	reference	0.99 (0.91, 1.08)	reference
		0-3 M	169	1.08 (1, 1.16)	*	1.03 (0.95, 1.12)	*
		3-12 M	58	1.12 (1.03, 1.21)	*	1.07 (0.98, 1.17)	*
eGFR (mL/min/1.73m^2^)	Sitagliptin				<.0001		<.0001
		baseline	831	74 (72.7, 75.4)	reference	74.4 (73.2, 75.5)	reference
		0-3 M	795	72.1 (70.8, 73.5)	*	72.4 (71.3, 73.6)	*
		3-12 M	198	70 (68.3, 71.7)	*	70.4 (68.8, 71.9)	*
	Vildagliptin				<.0001		<.0001
		baseline	233	70.3 (67.3, 73.3)	reference	70.6 (67.7, 73.6)	reference
		0-3 M	222	67.5 (64.5, 70.6)	*	67.8 (64.9, 70.8)	*
		3-12 M	60	69 (65.6, 72.4)		69.3 (66, 72.7)	
	Teneligliptin				<.0001		<.0001
		baseline	259	66.3 (63.6, 69)	reference	67 (64.4, 69.5)	reference
		0-3 M	239	63.6 (60.8, 66.3)	*	64.2 (61.7, 66.8)	*
		3-12 M	98	63 (60, 66)	*	63.7 (60.8, 66.5)	*
	Alogliptin				0.0009		0.0009
		baseline	226	72.5 (70, 75)	reference	73.2 (70.8, 75.7)	reference
		0-3 M	205	70.6 (68, 73.1)	*	71.3 (68.8, 73.8)	*
		3-12 M	62	70.4 (67.5, 73.3)		71.2 (68.3, 74.1)	
	Linagliptin				0.001		0.001
		baseline	174	61.8 (58, 65.7)	reference	62.2 (58.3, 66.1)	reference
		0-3 M	169	59.8 (56, 63.7)	*	60.2 (56.3, 64.1)	*
		3-12 M	58	59.5 (55.4, 63.6)	*	59.9 (55.7, 64)	*
HDL (mg/dL)	Sitagliptin				0.0037		0.0033
		baseline	726	51.3 (50.3, 52.3)	reference	52.2 (51.2, 53.2)	reference
		0-3 M	685	50.5 (49.4, 51.5)	*	51.4 (50.3, 52.4)	*
		3-12 M	141	50 (48.6, 51.5)		50.9 (49.5, 52.3)	*
	Vildagliptin				0.0007		0.0008
		baseline	203	47.3 (45.6, 49)	reference	48.6 (46.9, 50.3)	reference
		0-3 M	194	46.1 (44.4, 47.8)	*	47.4 (45.7, 49.1)	*
		3-12 M	50	44.5 (42.3, 46.6)	*	45.8 (43.7, 48)	*
	Teneligliptin				0.427		0.4293
		baseline	212	50.1 (48.3, 52)	reference	51.8 (49.9, 53.6)	reference
		0-3 M	188	50.2 (48.4, 52.1)		51.8 (50, 53.7)	
		3-12 M	78	49.3 (47.1, 51.4)		50.9 (48.7, 53)	
	Alogliptin				0.5739		0.5798
		baseline	183	47.5 (45.6, 49.5)	reference	48.6 (46.5, 50.7)	reference
		0-3 M	163	47.1 (45.1, 49)		48.1 (46, 50.2)	
		3-12 M	47	46.7 (44.2, 49.3)		47.8 (45.1, 50.4)	
	Linagliptin				0.3891		0.3894
		baseline	144	48.9 (46.8, 51)	reference	49.7 (47.5, 51.9)	reference
		0-3 M	140	48.3 (46.2, 50.4)		49.1 (46.9, 51.3)	
		3-12 M	43	49.7 (47, 52.3)		50.4 (47.7, 53.2)	
TC (mg/dL)	Sitagliptin				0.0004		0.0004
		baseline	728	192.3 (189.8, 194.8)	reference	194 (191.5, 196.5)	reference
		0-3 M	682	188.9 (186.3, 191.4)	*	190.6 (188, 193.2)	*
		3-12 M	161	194.3 (190.4, 198.3)		196 (192.1, 200)	
	Vildagliptin				0.0071		0.0024
		baseline	204	188.7 (183.6, 193.8)	reference	190.9 (185.6, 196.2)	reference
		0-3 M	194	183.4 (178.2, 188.6)	*	185.5 (180.2, 190.9)	*
		3-12 M	50	183.7 (176.5, 190.9)		186 (178.7, 193.4)	
	Teneligliptin				0.0668		0.0764
		baseline	217	190.1 (185.2, 195)	reference	193.6 (188.7, 198.5)	reference
		0-3 M	196	186.5 (181.5, 191.5)		189.9 (184.9, 194.9)	
		3-12 M	76	185 (178.5, 191.4)		188.9 (182.4, 195.3)	
	Alogliptin				0.043*		0.0372
		baseline	176	188 (182.5, 193.5)	reference	189.5 (183.6, 195.4)	reference
		0-3 M	153	183.4 (177.8, 189)	*	184.8 (178.8, 190.8)	*
		3-12 M	48	188.4 (180.9, 195.9)		190.1 (182.3, 197.9)	
	Linagliptin				0.1222		0.1105
		baseline	151	189.3 (183.1, 195.5)	reference	191.6 (185.2, 198)	
		0-3 M	146	186.3 (180.1, 192.5)		188.6 (182.2, 195)	
		3-12 M	50	191.4 (183.8, 198.9)		193.8 (186.1, 201.6)	
TG (mg/dL)	Sitagliptin				0.0473		0.0469
		baseline	755	145.1 (139.4, 150.7)	reference	142.9 (137.1, 148.8)	reference
		0-3 M	712	139.1 (133.3, 144.8)	*	136.9 (131, 142.8)	*
		3-12 M	160	141.9 (132.4, 151.4)		140 (130.4, 149.6)	
	Vildagliptin				0.0203		0.0072
		baseline	206	160.3 (148.9, 171.7)	reference	190.9 (185.6, 196.2)	reference
		0-3 M	195	148.2 (136.6, 159.7)	*	185.5 (180.2, 190.9)	*
		3-12 M	54	144.4 (126.8, 162)		186 (178.7, 193.4)	
	Teneligliptin				0.1054		0.1204
		baseline	233	151.3 (140.7, 161.9)	reference	149.4 (138.6, 160.3)	reference
		0-3 M	209	145.2 (134.2, 156.1)		143.1 (132, 154.3)	
		3-12 M	85	136.4 (121.3, 151.4)		135.2 (120, 150.4)	
	Alogliptin				0.0086		0.0077
		baseline	197	155 (143.6, 166.3)	reference	155.3 (143.1, 167.5)	reference
		0-3 M	174	140.3 (128.6, 152)	*	140.5 (128, 153)	*
		3-12 M	51	152.2 (135, 169.5)		153 (135.1, 170.9)	
	Linagliptin				0.9511		0.9521
		baseline	159	146.2 (134, 158.5)	reference	146.8 (134, 159.6)	reference
		0-3 M	154	144.5 (132.2, 156.9)		145.1 (132.1, 158)	
		3-12 M	49	144.6 (126.1, 163)		145.4 (126.5, 164.4)	
AST (U/L)	Sitagliptin				0.0879		0.0856
		baseline	830	27.8 (26.6, 29)	reference	27.5 (26.3, 28.7)	reference
		0-3 M	794	26.8 (25.6, 28.1)		26.5 (25.3, 27.8)	
		3-12 M	199	28 (26.2, 29.8)		27.7 (25.9, 29.5)	
	Vildagliptin				0.3947		0.3942
		baseline	232	30.3 (27.1, 33.5)	reference	30.5 (27.1, 33.9)	reference
		0-3 M	222	31.2 (28, 34.4)		31.4 (28, 34.8)	
		3-12 M	57	32.6 (28.2, 37)		32.8 (28.2, 37.4)	
	Teneligliptin				0.7444		0.7366
		baseline	256	29.8 (26.9, 32.8)	reference	30.6 (27.5, 33.6)	reference
		0-3 M	236	28.5 (25.5, 31.6)		29.2 (26.1, 32.4)	
		3-12 M	97	29.4 (24.9, 33.9)		30.2 (25.7, 34.8)	
	Alogliptin				0.0056		0.0056
		baseline	221	29.9 (27.3, 32.4)	reference	29.5 (26.7, 32.3)	Reference
		0-3 M	200	26.8 (24.2, 29.4)	*	26.4 (23.6, 29.2)	*
		3-12 M	61	28.4 (24.8, 32)		28 (24.3, 31.8)	
	Linagliptin				0.3101		0.3365
		baseline	176	24.7 (22.7, 26.7)	reference	25.1 (23.1, 27.1)	reference
		0-3 M	171	24.2 (22.2, 26.2)		24.6 (22.6, 26.7)	
		3-12 M	58	23.3 (20.9, 25.7)		23.8 (21.3, 26.3)	
ALT (U/L)	Sitagliptin				<.0001		<.0001
		baseline	830	30.4 (28.8, 32)	reference	29.8 (28.3, 31.4)	reference
		0-3 M	794	28.1 (26.5, 29.7)	*	27.5 (25.9, 29.1)	*
		3-12 M	199	27.8 (25.5, 30.1)	*	27.2 (25, 29.5)	*
	Vildagliptin				0.1288		0.1286
		baseline	233	32.3 (29.1, 35.6)	reference	32 (28.7, 35.2)	reference
		0-3 M	223	30.2 (27, 33.5)		29.8 (26.5, 33.2)	
		3-12 M	58	31.6 (27.2, 36)		31.1 (26.7, 35.6)	
	Teneligliptin				0.0824		0.089
		baseline	256	30.2 (27, 33.4)	reference	30.6 (27.3, 33.9)	reference
		0-3 M	236	27.5 (24.2, 30.8)		27.9 (24.5, 31.3)	
		3-12 M	97	25.5 (20.9, 30.2)		26 (21.3, 30.8)	
	Alogliptin				<.0001		<.0001
		baseline	223	34.7 (31.1, 38.3)	reference	34.1 (30.3, 37.9)	reference
		0-3 M	202	29 (25.4, 32.6)	*	28.4 (24.5, 32.2)	*
		3-12 M	61	29.6 (24.8, 34.5)	*	29.1 (24, 34.1)	*
	Linagliptin				<.0001		<.0001
		baseline	176	26.2 (22.6, 29.8)	reference	26.5 (23.2, 29.9)	reference
		0-3 M	171	23.4 (19.8, 26.9)	*	23.7 (20.3, 27.1)	*
		3-12 M	58	22.4 (18.4, 26.3)	*	22.8 (19, 26.6)	*

*p < 0.05 (compared with baseline period, multiple-comparison test: Dunnett’s post-hoc analysis), ^a^Adjusted for age and sex

*LS mean* least square mean, *CI* confidence interval, *HbA1c* hemoglobin A1c, *eGFR* estimated glomerular filtration rate, *HDL* high density lipoprotein, *TC* total cholesterol, *TG* triglyceride, *AST* aspartate aminotransferase, *ALT* alanine aminotransferase

Table 6 shows the least square mean changes in laboratory parameters during the exposure period from baseline. After adjustment, there was no significant difference in mean changes in concentrations of laboratory parameters among the five groups of DPP-4 inhibitor users.

**Table 6:** Comparison of mean changes in laboratory parameters from baseline during exposure periods among five DPP-4 inhibitors

Laboratory parameters	Drugs	Unadjusted LS mean (95%CI)		*p*-value	^b^Adjusted LS mean (95%CI)		*p*-value
		3 M	12 M		3 M	12 M	
^a^HbA1c (%)				0.001			0.0668
	Sitagliptin	−0.46 (−0.52, − 0.4)	− 0.55 (− 0.65, − 0.45)		− 0.46 (− 0.53, − 0.38)	− 0.6 (− 0.71, − 0.49)	
	Vildagliptin	− 0.61 (− 0.72, − 0.5)	−0.69 (− 0.87, − 0.51)		−0.6 (− 0.7, − 0.5)	−0.64 (− 0.81, − 0.47)	
	Teneligliptin	− 0.64 (− 0.75, − 0.53)	− 0.9 (− 1.05, − 0.74)		− 0.54 (− 0.64, − 0.43)	− 0.76 (− 0.91, − 0.62)	
	Alogliptin	− 0.43 (− 0.55, − 0.32)	− 0.58 (− 0.77, − 0.39)		− 0.57 (− 0.68, − 0.47)	−0.74 (− 0.91, − 0.56)	
	Linagliptin	− 0.49 (− 0.62, − 0.36)	−0.59 (− 0.78, − 0.4)		−0.58 (− 0.7, − 0.46)	−0.69 (− 0.87, − 0.51)	
^a^creatinine (mg/dL)				0.0259			0.1505
	Sitagliptin	0.02 (0.01, 0.03)	0.06 (0.04, 0.08)		0.04 (0.02, 0.06)	0.08 (0.05, 0.11)	
	Vildagliptin	0.02 (0, 0.05)	0.04 (0, 0.08)		0.04 (0.01, 0.06)	0.06 (0.02, 0.1)	
	Teneligliptin	0.04 (0.02, 0.06)	0.11 (0.08, 0.14)		0.05 (0.02, 0.07)	0.12 (0.08, 0.15)	
	Alogliptin	0.03 (0, 0.05)	0.04 (0, 0.08)		0.04 (0.02, 0.07)	0.05 (0.01, 0.1)	
	Linagliptin	0.04 (0.02, 0.07)	0.07 (0.03, 0.12)		0.05 (0.02, 0.08)	0.07 (0.03, 0.12)	
^a^eGFR (mL/min/1.73m^2^)				0.4528			0.4143
	Sitagliptin	− 1.92 (− 2.55, − 1.29)	−4.13 (− 5.27, − 2.98)		− 2.9 (− 3.86, − 1.93)	− 4.9 (− 6.25, − 3.54)	
	Vildagliptin	− 2.79 (− 3.98, − 1.6)	− 1.33 (− 3.42, 0.76)		− 3.97 (− 5.31, − 2.64)	− 2.36 (− 4.54, − 0.18)	
	Teneligliptin	− 2.73 (− 3.88, − 1.59)	− 3.22 (− 4.88, − 1.56)		− 4.23 (− 5.57, − 2.89)	−4.68 (− 6.48, − 2.87)	
	Alogliptin	− 1.99 (− 3.23, − 0.76)	−1.94 (− 4.03, 0.14)		− 2.96 (− 4.35, − 1.57)	− 3.01 (− 5.17, − 0.84)	
	Linagliptin	− 2 (− 3.36, − 0.63)	−2.24 (− 4.37, − 0.1)		− 3.85 (− 5.35, − 2.35)	− 4.13 (− 6.35, − 1.9)	
^a^HDL (mg/dL)				0.1268			0.0613
	Sitagliptin	− 0.87 (− 1.42, − 0.31)	−1.33 (− 2.45, − 0.21)		− 0.8 (− 1.65, 0.05)	−1.37 (− 2.65, − 0.09)	
	Vildagliptin	− 1.15 (− 2.19, − 0.1)	−2.9 (− 4.78, − 1.03)		−1.67 (− 2.85, − 0.49)	−3.29 (− 5.23, − 1.35)	
	Teneligliptin	0.1 (− 0.95, 1.15)	− 0.9 (− 2.44, 0.63)		0.1 (−1.12, 1.31)	− 0.93 (− 2.58, 0.71)	
	Alogliptin	− 0.46 (− 1.59, 0.67)	− 0.89 (− 2.86, 1.08)		− 0.93 (− 2.2, 0.33)	−1.33 (− 3.36, 0.7)	
	Linagliptin	−0.57 (−1.8, 0.66)	0.84 (− 1.18, 2.86)		− 0.87 (− 2.2, 0.46)	0.36 (− 1.71, 2.43)	
^a^TC (mg/dL)				0.1469			0.3631
	Sitagliptin	− 3.4 (− 5.3, −1.51)	1.3 (− 2.1, 4.7)		− 3.14 (− 6.14, −0.14)	1.06 (− 3.05, 5.16)	
	Vildagliptin	− 5.22 (− 8.79, − 1.65)	− 6.57 (− 12.63, − 0.5)		− 4.67 (− 8.81, − 0.53)	− 6.05 (− 12.51, 0.42)	
	Teneligliptin	−3.22 (− 6.74, 0.3)	− 6.56 (− 11.63, − 1.49)		− 1.71 (− 5.89, 2.48)	− 5 (− 10.57, 0.57)	
	Alogliptin	−4.58 (− 8.56, − 0.6)	−0.18 (− 6.63, 6.27)		−4.32 (− 8.84, 0.19)	−0.36 (− 7.17, 6.46)	
	Linagliptin	−2.91 (− 7.03, 1.21)	1.21 (− 4.92, 7.33)		− 2.76 (− 7.35, 1.83)	1.12 (− 5.38, 7.62)	
^a^TG (mg/dL)				0.4256			0.536
	Sitagliptin	−5.96 (− 10.95, −0.97)	− 3.1 (− 12.32, 6.13)		− 1.49 (− 9.42, 6.43)	0.92 (− 10.15, 12)	
	Vildagliptin	−12.01 (−21.55, − 2.47)	−15.02 (− 30.92, 0.89)		− 7.39 (− 18.46, 3.68)	− 10.3 (− 27.3, 6.69)	
	Teneligliptin	− 6.32 (− 15.46, 2.82)	− 12.27 (− 25.28, 0.74)		0.5 (− 10.55, 11.55)	− 5.66 (− 20.13, 8.8)	
	Alogliptin	−14.92 (− 24.95, − 4.9)	−2.56 (− 19.42, 14.29)		−10.28 (− 21.97, 1.41)	1.49 (− 16.39, 19.36)	
	Linagliptin	−1.78 (− 12.55, 8.99)	1.45 (− 15.24, 18.14)		2.27 (− 9.79, 14.34)	5.12 (− 12.56, 22.8)	
^a^AST (U/L)				0.1086			
	Sitagliptin	−0.97 (−2.06, 0.13)	− 0.27 (− 2.41, 1.86)		−2.65 (− 4.25, −1.06)	−2.02 (− 4.45, 0.4)	0.105
	Vildagliptin	0.91 (−1.16, 2.98)	3.11 (− 0.89, 7.1)		0.05 (−2.2, 2.29)	0.57 (− 3.47, 4.62)	
	Teneligliptin	−1.43 (− 3.44, 0.57)	− 0.41 (− 3.48, 2.67)		− 2.32 (− 4.58, − 0.07)	− 1.44 (− 4.66, 1.77)	
	Alogliptin	− 2.87 (− 5.04, − 0.69)	−2.24 (− 6.11, 1.64)		− 4.23 (− 6.6, − 1.86)	−2.73 (− 6.64, 1.17)	
	Linagliptin	−0.49 (−2.84, 1.87)	− 1.23 (− 5.19, 2.73)		− 2.99 (− 5.48, − 0.5)	−3.93 (− 7.94, 0.09)	
^a^ALT (U/L)				0.1576			0.2768
	Sitagliptin	−2.33 (−3.56, − 1.11)	− 1.92 (− 3.99, 0.16)		−4.08 (− 5.82, − 2.34)	−3.74 (− 6.15, − 1.32)	
	Vildagliptin	-2.05 (-4.36, 0.26)	−0.25 (− 4.1, 3.6)		−2.88 (− 5.29, − 0.47)	−1.81 (− 5.75, 2.12)	
	Teneligliptin	− 2.74 (− 4.97, − 0.51)	− 3.69 (− 6.77, − 0.6)		− 3.73 (− 6.14, − 1.31)	− 3.42 (− 7.28, 0.44)	
	Alogliptin	−5.69 (− 8.1, − 3.28)	−5.08 (− 8.94, − 1.23)		−6.13 (− 8.66, − 3.6)	−4.86 (− 8.76, − 0.97)	
	Linagliptin	−2.83 (− 5.47, − 0.18)	−3.68 (− 7.57, 0.22)		−5.15 (− 7.82, − 2.48)	−6.19 (− 10.13, − 2.24)	

^a^indicates change in laboratory parameter during exposure period from baseline

*HbA1c* hemoglobin A1c, *eGFR* estimated glomerular filtration rate, *HDL* high density lipoprotein, *TC* total cholesterol, *TG* triglyceride, *AST* aspartate aminotransferase, *ALT* alanine aminotransferase, *LS mean* least square mean, *CI* confidence interval, *p* value: *p* value among five DPP-4 inhibitors (multiple-comparison test)

^b^Adjusted for time, age, sex, medical history in baseline period including ischemic heart disease and hypertension, medication in baseline period including hypoglycemic drugs and lipid-lowering drugs, baseline concentration of HbA1c, and baseline concentration of each parameter

## Discussion

In this study, we compared the long-term effect of monotherapy among five DPP-4 inhibitors, sitagliptin, vildagliptin, teneligliptin, alogliptin, and linagliptin, on laboratory parameters in patients with type 2 DM, during 12 months of treatment. Our results showed a favorable effect on HbA1c concentration in users of five DPP-4 inhibitors, a slightly unfavorable effect on serum creatinine concentration in users of five DPP-4 inhibitors, a favorable effect on lipid metabolism in sitagliptin, vildagliptin, and alogliptin users, and a favorable effect on hepatic parameters in sitagliptin, alogliptin, and linagliptin users. However, there was no significant difference in the mean change in concentration of any laboratory parameter among the five groups of DPP-4 inhibitor users.

DPP-4 inhibitors are known to lower glycemic parameters in a glucose-dependent manner [2, 5, 6]. Compared with placebo, DPP-4 inhibitor monotherapy and combination with other agents significantly decreased HbA1c concentration at 24 weeks by 0.6% [5]. The mean reduction in HbA1c concentration was similar to that with DPP-4 inhibitors in patients with renal dysfunction [16]. In our study, the HbA1C concentration during the exposure period was significantly lower than that in the baseline period in all DPP-4 inhibitor users, and the mean change in HbA1c concentration showed no significant difference among the five groups of DPP-4 inhibitor users. These results confirmed that these five DPP-4 inhibitors are effective for glycemic control.

Serum creatinine concentration was increased during the exposure period compared with that in the baseline period in all DPP-4 inhibitor users. However, the mean change in creatinine concentration showed no significant difference among the five groups of DPP-4 inhibitor users. eGFR was significantly decreased in the exposure period in patients with sitagliptin, vildagliptin, teneligliptin, and linagliptin, and significantly decreased during 3 months in patients with alogliptin. However, the mean change in eGFR showed no significant difference among the five groups of DPP-4 inhibitor users. Sitagliptin has been reported to decrease eGFR in patients with diabetes mellitus with baseline eGFR > 60 up to 12 months [17]. The decrease in eGFR is explained by elimination of hyperfiltration. Treatment with GLP-1 has been reported to increase sodium excretion, and, via tubulo-glomerular feedback, decrease GFR in insulin-resistant obese men [18]. Therefore, our result of an increase in serum creatinine concentration in users of the five DPP-4 inhibitors might reflect the elimination of hyperfiltration, and the effect may be small and may not be of clinical concern, consistent with previous reports.

Serum HDL concentration during the exposure period was significantly lower than that in the baseline period in sitagliptin and vildagliptin users. Serum TC and TG concentrations during the 3 M period were significantly lower than that in the baseline period in sitagliptin, vildagliptin, and alogliptin users. However, the mean changes in HDL, TC, and TG concentrations showed no significant difference among the five groups of DPP-4 inhibitor users. The results of DPP-4 inhibitors’ effects on lipid metabolism parameters are diverse and inconclusive [2]. Park et al. reported a significant increase in HDL concentration in patients with 12-week administration of sitagliptin or linagliptin, and showed a decreasing trend in TC, TG, and LD in patients with 12-week administration of sitagliptin, vildagliptin, or linagliptin [19]. Kubota et al. reported that administration of sitagliptin significantly decreased TC and LDL concentrations, and tended to decrease HDL concentration in patients with type 2 DM, up to 12 weeks [9]. Takeda et al. reported that administration of alogliptin significantly decreased TC and LDL concentrations in patient with type 2 DM, up to 12 weeks [20]. In our results, HDL concentration was slightly decreased in the exposure period in sitagliptin and vildagliptin users. TC and TG concentrations were significantly decreased in the 3 M period in sitagliptin, vildagliptin, and alogliptin users. However, the mean changes in HDL, TC, and TG concentrations showed no significant difference among the five groups of DPP-4 inhibitor users. DPP-4 inhibitors might have a beneficial effect on lipid concentrations; however, further studies are needed to investigate the mechanism of the effect of DPP-4 inhibitors on lipid metabolism [2].

Of the hepatic parameters in our results, serum AST concentration during the 3 M period was significantly lower than that in the baseline period in alogliptin users. Serum ALT concentration during the exposure period was significantly lower than that in the baseline period in sitagliptin, alogliptin, and linagliptin users. However, the mean changes in AST and ALT concentrations showed no significant difference among the five groups of DPP-4 inhibitor users. Kusunoki et al. previously reported that serum AST, ALT, and γ-GT concentrations were significantly decreased in patients with 6-month administration of combination therapy with a DPP-4 inhibitor and a sodium glucose co-transporter 2 (SGLT2) inhibitor [21]. Aoki et al. previously reported that serum ALT and γ-GT concentrations were significantly decreased in patients with 16-week administration of combination therapy with alogliptin and pioglitazone compared with those with monotherapy [22]. Twelve-month treatment with alogliptin was previously reported to decrease NAFLD score [23]. Considering these results, some DPP-4 inhibitors might have a beneficial effect on hepatic metabolism; however, further studies are needed to investigate the mechanism of the effect of DPP-4 inhibitors on hepatic parameters.

### Limitations

There are several limitations of our study. First, there is a possibility of selection bias and confounding factors because this study was a retrospective study using non-randomized data. We applied a multivariate regression model, which enabled us to control for potential confounding variables among the five DPP-4 inhibitor groups; however, their ability to control for differences was limited to available or measurable variables. Second, we did not exclude patients who had received other antidiabetic drugs before the initiation of DPP-4 inhibitors, because DPP-4 inhibitors are relatively new drugs and many patients (about 44 to 71%) were then treated with other antidiabetic drugs, including insulin and oral hypoglycemic drugs. Sulphonylureas and thiazolidinediones are known to be associated with an increase in body weight [24]. Metformin, one of the biguanides, is reported to improve liver enzymes in patients with NASH [25]. We used rigorous statistical methods to control for differences in prior treatment among the five DPP-4 inhibitor groups; however, we would like to study patients firstly treated with antidiabetic drugs when a sufficient sample has been collected. Third, the standard dose of sitagliptin in Japan is 50 mg, which is half the world standard dose; this is because, in Japanese cases, it has been reported that there are no statistically significant differences in HbA1c, fasting plasma glucose, and 2 h postprandial glucose concentration among doses of sitagliptin of 50, 100, and 200 mg [26]. Therefore, the adverse effects of sitagliptin might have been mitigated at this dose. Fourth, the number of sitagliptin users was larger than those of the other DPP-4 inhibitors. Because sitagliptin was the first entrant and is the dominant DPP-4 inhibitor in Japan, this unbalanced sample size might have reflected the market share of sitagliptin in Japan, suggesting that this study is a good reflection of clinical practice. We used a mixed linear model, which is a rigorous statistical model that enables adjustment for unbalanced sample sizes. The findings of our study are relevant to clinical practice in real world settings, and have sufficient reliability based on a sophisticated statistical method, however, further studies such as randomized clinical trials will be needed for confirmation.

## Conclusion

In this study, we showed the effect among five DPP-4 inhibitors on glycemic, renal, and lipid metabolism, and hepatic parameters. DPP-4 inhibitors are well-tolerated hypoglycemic drugs; however, physicians should monitor laboratory parameters for at least 12 months after their initiation.

## Data Availability

The datasets generated and analyzed during the current study are not publicly available because approval was not obtained for the sharing of subject data from the Ethical Committee of NUSM. Data are however available from the corresponding author upon reasonable request and with permission of the Ethical Committee of NUSM.

## Abbreviations

ALT: Alanine aminotransferase
AST: Aspartate aminotransferase
CDW: Clinical Data Warehouse
CYP: Cytochrome P450
eGFR: Estimated glomerular filtration rate
DM: Diabetes mellitus
DPP-4: Dipeptidyl-peptidase 4
HbA1c: Hemoglobin A1c
HDL: High density lipoprotein
GLP-1: Glucagon-like peptide-1
NAFLD: Nonalcoholic fatty liver disease
NASH: Nonalcoholic steatohepatitis
NUSM: Nihon University School of Medicine
TC: Total cholesterol
TG: Triglyceride
